# A Swedish Haplotype GWAS in Familial and Sporadic Site-Specific Colorectal Cancer

**DOI:** 10.3390/ijms27062758

**Published:** 2026-03-18

**Authors:** Litika Vermani, Shabane Barot, Annika Lindblom

**Affiliations:** 1 Department of Molecular Medicine and Surgery, Karolinska Institutet, 171 76 Stockholm, Sweden; litikavermani@cacharcancerhospital.org; 2 Department of Clinical Genetics and Genomics, Karolinska University Hospital, 171 64 Stockholm, Sweden; 3 Dr. S Krishnamurthi Centre for Research and Education in Cancer, Cachar Cancer Hospital and Research Centre, Silchar 788015, India; 4 Department of Clinical Science and Education, Södersjukhuset, Karolinska Institutet, 118 83 Stockholm, Sweden; shabane.barot@ki.se; 5 Department of Oncology, Södersjukhuset, 118 83 Stockholm, Sweden

**Keywords:** colorectal cancer, genome-wide association study, risk, site-specific, location, GWAS, predisposition, cecum, right-sided, left-sided

## Abstract

Genetic variants specific to anatomical subsites of colorectal cancer are known to play a crucial role in its prognosis and treatment. We undertook a haplotype-based genome-wide association study (GWAS) to identify specific genetic risk loci for three sites: cecum, right colorectum, and left colorectum. Six different haplotype GWAS were performed using familial and sporadic colorectal cancer cases with tumors at three different sites. The studies included 2358 CRC cases and 1642 healthy controls. A logistic regression model using PLINK v.1.07 software was employed, and risk loci with a *p*-value of 5 × 10^−8^ were considered statistically significant. In total, 29 distinct risk loci were identified in the analyses of familial and sporadic cases of cecal and proximal colon cancer. The results from the analyses of familial and sporadic left-sided colorectal cancer did not meet the strict criteria for significance. Among the loci that were associated with cecal cancer, 14 were familial, and seven were sporadic. Among the other right-sided colon cancer loci, six were familial, and two were sporadic. Coding genes were found at 18 of the 29 loci. Our findings of site-specific genetic risk loci support the growing evidence for divergent pathways in familial and sporadic colorectal cancer across different colorectal sites. The data support a model where the rise in proximal tumors, both familial and sporadic, is influenced by genetic risk to a higher degree than that of distal tumors. These findings are important for understanding colorectal carcinogenesis and could, after future studies, lead to new applications in cancer prevention, treatment, and prognosis.

## 1. Introduction

There is a global increase in the incidence of colorectal cancer (CRC), especially in early-onset disease, and a decrease in CRC-related mortality [1]. Approximately 5–6% of all CRC cases have a germline mutation in a known high-penetrance cancer gene, while most others are hypothesized to arise due to multiple genetic, environmental, and lifestyle factors that are characteristic of complex disease. Right- and left-sided colorectal tumors differ in embryologic origin, morphology, histology, and molecular profiles. Right-sided (proximal) colon tumors (RCC) are derived from the midgut, whereas left-sided (distal) tumors (LCC) are derived from the hindgut [2]. Accumulating evidence suggests that proximal and distal tumors have distinct clinical characteristics and differences in prognosis and treatment outcomes [3]. There are also differences in pathology. Serrated adenomas are more commonly found in the right colon, whereas RCCs tend to be flatter, of a mucinous phenotype, with increased T-cell infiltration and are more often diploid- and microsatellite-unstable. In contrast, distal tumors often demonstrate aneuploidy and chromosomal instability, as well as tubular and villous adenocarcinomas and a polypoid-like morphology [3]. The differences extend to the prognosis, the treatment response, and the preferred metastatic sites of disease, and there is a clear reduced risk of death in left-sided CRC as compared to right-sided, regardless of the disease burden and known mutation status [4]. These divergent patterns may be partly explained by the distinct embryonic origins of the right and left colon, which contribute to their molecular and biological heterogeneity.

Given these differences in pathways and somatic genetics, it is plausible that predisposing genetic variants that increase CRC risk also vary by tumor location. A recent GWAS used a dataset of single variants from 48,214 CRC cases and 64,159 controls to conduct five genome-wide SNP-association scans of case subgroups that were defined by the location of their primary tumors within the colorectum [5]. Thirteen loci, not reported by previous GWAS for overall CRC risk, reached genome-wide significance (*p* < 5 × 10^−8^) [5]. Distinct loci were found in four of the analyses: three for tumors in the colon, one for tumors in the rectum, three for tumors in the proximal colon, and six for distal tumors. These findings suggested a heterogeneity in risk loci among anatomical tumor subsites [5].

We aimed to investigate whether genetic predisposition varies across anatomical locations within the colorectum using a different method. We have, in our previous studies, seen that sliding-window haplotype GWAS can find rarer loci with higher odds ratios compared to SNP GWAS, which is why we chose this study design in this study. The same microchip was used as in the paper by Huyghe et al. mentioned above [5]. We conducted six site-specific haplotype GWAS, comparing familial and sporadic cases with tumors in the cecum and other right-sided (RCC) or left-sided (LCC) locations, using the same healthy controls.

## 2. Results

**Figure 1:**
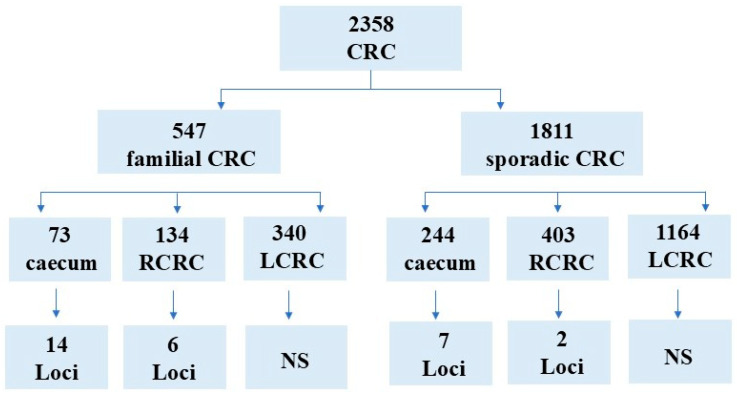
Six GWAS in familial and sporadic colorectal cancer patients in three tumor locations.

Six haplotype GWAS were performed in patients with familial and sporadic diseases and tumors that originated in the cecum, RCC, or LCC (Figure 1). Several, mostly novel, loci were identified, and all but one contained coding genes and/or RNA genes within the haplotype boundaries. Protein-coding genes were considered the most likely to act as targets. All PLINK analyses used GRCh37, as indicated in all Supplementary Tables. The data have been updated to GRCh38 in tables.

### 2.1. Analyses in Cases with Tumors in the Cecum

The GWAS of 73 familial cases with cecal cancer resulted in fourteen significant loci with ORs between 4.4 and 22.9 (Supplementary Table S1; Table 1). Eight of the fourteen loci contained genes, five had one or more RNA genes, and one haplotype had no gene. The GWAS of 244 patients with sporadic cecal tumors identified seven significant haplotypes, with ORs ranging from 1.8 to 6.69 (Supplementary Table S2; Table 1). Two of the seven loci had coding genes within their haplotype regions.

### 2.2. Analyses in Cases with RCC

In the analysis of 134 familial RCC cases versus healthy controls, six haplotypes at six distinct loci were significantly associated with disease (Supplementary Table S3; Table 2). The ORs ranged from 4.29 to 8.68 (Table 2). All the loci contained one or several coding genes. The analysis of 403 sporadic RCC cases versus healthy controls identified two significant loci with coding genes, with ORs ranging from 4.32 to 5.13 (Supplementary Table S4; Table 2).

### 2.3. Analyses in Cases with LCC

None of the analyses in LCC (340 familial and 1164 sporadic cases) reached statistical significance (*p* < 5 × 10^−8^) (Supplementary Tables S5 and S6).

## 3. Discussion

Six haplotype-based GWAS of CRC cases were conducted. The samples were stratified by family history and anatomical subsite. The findings indicate that different sites in familial and sporadic colorectal cancer are associated with distinct predisposing loci and genes. Our results support the growing evidence that proximal and distal CRCs are biologically heterogeneous rather than a uniform disease. The highest ORs were observed at unique loci in the analysis of familial cases with cecal cancer. The differences among sites in the colorectum reflect the differences in embryological origin and exposure to environmental and lifestyle factors [2,3,4]. The current haplotype GWAS identified significant loci that were confined to cecal and right-sided colon cancer, whereas no significant risk loci were detected in either familial or sporadic left-sided colon or rectal cancer using the strict *p*-value for significance. This indicated that germline genetic predisposition plays a greater role in the development of cecal tumors and right-sided colon cancers than in left-sided colorectal cancers. It also supports the general opinion that most sporadic cases develop in the left colon [6]. In addition, the analysis of sporadic LCC identified more loci in the sporadic cases than in the familial cases, as opposed to the analyses in cases with proximal tumors. This confirms the finding that left-sided tumors tend to be more associated with environmental influence and genetic modifiers. There were many RNAs suggested in the analyses of both the cecum and RCC. Regulatory non-coding RNA influences cell physiopathology and modulates cells by regulating gene expression in different ways [7].

One limitation of this study is that the six studies used small and disproportionate sample sizes. In particular, the loci with the highest ORs had the fewest cases, yet these remained sufficient for statistically significant results. Another possible limitation is that a quite large cohort of the same controls was used in all analyses. Still, many statistically significant loci were also identified in the smaller cohorts, and it is unclear whether this could have influenced the results negatively. The distribution of risk variants might not strictly follow the sites that were chosen for analysis, but it could relate to a gradient from the cecum to the rectum with a decreasing number of high-risk variants and an increasing number with a lower risk. It is difficult to determine what *p*-value criteria should be used in the haplotype analysis, and suggestions to use both more strict (because of the many tests) and more loose criteria (because they are not independent tests but rather testing the same locus numerous times) have been made. Therefore, we have chosen the *p*-value that is generally accepted for GWAS. Furthermore, the most important result of this paper is the differences in risk genes (loci) suggested between familial and sporadic tumors at different locations, rather than the exact *p*-value for statistical significance in each analysis.

The genes suggested in this study were compared to genes from previously published SNP-GWAS [8,9,10,11,12,13,14,15,16,17,18,19,20,21,22,23,24,25,26,27,28,29,30,31,32,33,34]. Only two genes, *ERGIC1* and *PITX1*, have previously been suggested as risk loci [32,34]. The loci identified in haplotype GWAS are often rare, while those identified by SNP GWAS are common; thus, none of the rare loci in the present study overlapped with the loci suggested by Huyghe et al. [5].

In our study, the ORs for the analysis in cases with cecal cancer were the highest, reaching 22.9 for the *WWTR1* locus. The *WWTR1* gene has been suggested to act as an oncogene, playing a crucial role in the proliferation of colorectal cancer cells and in tumor growth in vivo [35]. The current haplotype GWAS generated numerous loci and suggested many genes. Most of these loci contained a single gene, strongly suggesting that a risk variant at this locus was implicated in CRC, and many have already been reported in relation to CRC. The analysis of the patients with cecal tumors identified 21 loci: 20 of these were coding, or RNA genes, and nine of the ten contained only one gene. Six of these nine genes were already implicated in cancer. The *RYR2* gene is frequently mutated in CRC; in one study, it was among eight CRC-associated genes with mutation rates exceeding 20% [36]. *TRIM32* is a crucial member of the TRIM family, is highly expressed in numerous human cancers, and is associated with a poor prognosis. However, the mechanism of *TRIM32* in CRC is still unclear [37]. One study analyzed 54 commonly differentially expressed genes and found that genes, including *ARSJ*, were associated with CRC’s overall survival [38].

The RCC analysis identified eight significant loci: six in familial patients and two in sporadic patients. All eight had at least one coding gene, and five of these had genes previously associated with CRC. Two genes, *KIAA40* and *ABCA12*, were reported in a previous haplotype-based GWAS. *KIAA40* was suggested to act as a modifier gene in a subset of CRC cases selected because they reported smoking as a risk factor [39]. *ABCA12* was suggested as a modifying risk locus in CRC cases selected for physical inactivity in the same study [39]. Another published paper reported that the *KAT2B* gene decreased *BRCA2* expression in CRC and suggested that *KAT2B* acted on the PARPi response by regulating the expression of *BRCA2* [40]. The *RBM47* gene has been suggested to have an anti-tumor function [41]. The same gene was also found as one of ten risk loci in our previous haplotype GWAS of CRC patients with familial gastric and/or prostate cancer [42]. In that study, locus *RBM47* had a much lower OR (2.4) and a less significant *p*-value (*p* < 4.3 × 10^−6^) than in this study (OR = 7.95, *p* < 3.75 × 10^−8^) [42]. Fas and Fas ligand (FasL) are implicated in programmed cell death of apoptosis [43]. Cancer stem-like cells (CSCs) are proposed to act within tumor growth and relapse and are a target for cancer therapy. Aspirin was suggested to eliminate CSCs by a unique pathway (p300-Ach3K9-FasL) axis, which could explain the therapeutic significance of aspirin [44]. The gene *FLI1* has been implicated in CRC. DNA methyltransferase 3b (*DNMT3b*) was found to be significantly overexpressed in CRC, and low *DNMT3b* expression was associated with prolonged survival [45]. The inhibition of *DNMT3b* increased *FLI1* expression and inhibited the malignant phenotype of CRC cells. The inhibition of *FLI1* reversed phenotypic modulation by *DNMT3b* depletion in vitro and in vivo. It was suggested that *DNMT3b* potentiates CRC cell proliferation, migration, and invasion by downregulating *FLI1* [45].

In the GWAS comparing LCC with healthy controls, no loci met the strict criteria for statistical significance. However, numerous genes were suggested in both familial and sporadic analyses, and even if none of the loci reach the criteria for statistical significance, it is still possible that some could be of importance as modifier genes, and further studies are warranted before ruling them out.

## 4. Materials and Methods

### 4.1. Cases and Controls

Colorectal cancer cases were recruited as part of the Colorectal Cancer Low-risk study [46]. All of the newly diagnosed colorectal cancer cases from 14 hospitals in mid-Sweden were invited to participate. Blood samples were collected from participants between 2004 and 2009. The criteria for case inclusion and exclusion are detailed in a previous paper [47]. In the present study, we used 2358 CRC cases, 547 of which had a family history of CRC in at least one close relative, and 1811 of which were sporadic CRC cases, lacking a family history of CRC. In total, 1642 healthy men and women from the same Swedish geographical area served as controls in all six analyses. The controls consisted of 1106 healthy blood donors and 536 spouses without a family history of cancer. RCC tumors were defined as those located in the ascending colon, the right flexure, and the transverse colon. LCC tumors were defined as those occurring in the splenic flexure, descending colon, sigmoid colon, and rectum. The demographic and clinical features of the CRC cases that were selected for this study are described in detail in Table 3.

### 4.2. Genotyping, Quality Control

Peripheral blood was used for DNA extraction according to the standard procedures. The genotyping for both cases and controls was performed at the Center for Inherited Disease Research at Johns Hopkins University, US, using the Illumina Infinium^®^ OncoArray-500K (Illumina, San Diego, CA, USA) [28]. The first and second quality controls were performed within the CORECT (http://epi.grants.cancer.gov/gameon/ (accessed on 6 March 2026)) consortium and at Karolinska Institutet [28,47].

### 4.3. Haplotype Analysis and Statistics

Six haplotype association analyses were conducted using the software PLINK v.1.07 [48]. We employed a sliding-window approach, moving windows of predefined lengths from 1 to 25 SNPs across the genotyped loci in the 5′ to 3′ direction [48]. As information on chromosome phasing is lacking in genotype data, PLINK v.1.07 applies the expectation-maximization algorithm to estimate the haplotype frequencies within each window through statistical inference [49]. The population frequency (F) is an estimation based on the number of samples (cases and controls) used for each haplotype. All possible haplotypes within each window are tested. PLINK further investigates associations between the estimated haplotypes and CRC via logistic regression. The default minimum haplotype frequency cutoff of 0.01 was applied, excluding haplotypes with a frequency below 1% from individual testing and grouping them as a single rare category. All estimated haplotypes within each window were tested, with an arbitrarily selected haplotype serving as the reference [50]. PLINK provided ORs, Wald test statistics (squared t), and *p*-values for each haplotype. All of the analyses used the GRCh37 genome build. We applied the established genome-wide significance threshold for SNP GWAS (*p* < 5 × 10^−8^) [51]. The analysis involves multiple tests of each SNP. If all SNPs had two possible genotypes, a total of 2^50^ possible haplotypes would be generated. However, typically, the number of generated haplotypes for each locus is less than 25. Using haplotype windows of up to 25 SNPs thus generates several haplotypes representing the same unique region, each varying in length from 1 to 25 SNPs. This means that the number of haplotypes generated per SNP varies with SNP variability across haplotypes. This is described in detail for the first significant locus on chromosome 1 in the analysis of the patients with familial cecal cancer, in Supplementary Table S1 and Table 4a,b. Table 4a shows all haplotypes with *p* > 2.5 × 10^−6^ and Table 4b shows all haplotypes using all suggested haplotypes regardless of the *p*-value. Variants (SNPs) of the same sequence are observed across many of the generated haplotypes with various and less stringent *p*-values, all in bold in Table 4a,b. More detailed results from PLINK at this locus are presented in Table 4b, which illustrates all haplotypes with even less stringent *p*-values to represent the locus. Only the haplotype with the best *p*-value among all of the haplotypes representing the same haplotype at each locus is selected and presented as one locus under Results. Thus, in the examples in Table 4a,b, the haplotype TAGACAG at the position Chromosome 1: 237,203,483–237,257,650 (GRCh37) corresponds to this locus in Table 1 (the positions here have been converted to GRCh38). Due to the substantial computational demands, the analyses were done on high-performance computers at the UPPMAX Bianca cluster, which is part of the Uppsala Multidisciplinary Center for Advanced Computational Science.

## 5. Conclusions

Cancers arising in different sides of the colon are known to have very different biology and outcomes. Our haplotype-based GWAS revealed site-specific genetic risk profiles and genes in both familial and sporadic colorectal cancer, highlighting the importance of anatomical context in understanding the tumor initiation and progression at these sites. No risk loci reached statistical significance in LCC, suggesting that more important genetic risk factors are present in cases of cancer arising in the proximal colon. Our findings of new cancer genes may, after future studies, lead to new knowledge and personalized approaches in CRC risk assessments, treatment strategies, or prognostic predictions.

## Figures and Tables

**Table 1:** Unique statistically significant haplotypes in the analysis of familial and sporadic patients with cecum cancer.

Locus	BP1–BP2 (GRCh 38)	F	OR	*p*-Value	Genes in Familial Cecum Cases
1q43	237,040,183–237,094,350	0.02	11.00	1.25 × 10^−8^	*RYR2*
2p24.1	22,462,218–22,669,766	0.03	5.15	7.28 × 10^−9^	3 RNA genes
3q25.1	149,560,258–149,645,721	0.01	22.90	7.97 × 10^−9^	*WWTR1*
4q35.1	186,138,409–186,226,917	0.05	4.95	6.61 × 10^−9^	*FAM149A*, *CYP4V2*
4q28.3	135,767,281–135,921,964	0.03	5.98	9.51 × 10^−9^	2 RNA genes
4q28.3	135,591,600–135,732,439	0.02	7.54	2.77 × 10^−8^	no gene
4q26	113,912,134–114,083,870	0.01	11.40	3.19 × 10^−8^	*ARSJ*
5q35.1	172,796,042–172,911,613	0.03	6.22	4.6 × 10^−8^	*ERGIC1*
5q31.1	135,255,747–135,266,018	0.04	4.40	4.89 × 10^−8^	*PITX1*
7p14.3	29,608,046–29,805,092	0.01	10.20	7.51 × 10^−9^	4 RNA genes
8q24.3	141,758,131–141,842,023	0.01	13.10	6.51 × 10^−9^	RNA gene
8q24.22	133,327,751–133,389,266	0.01	14.40	3.69 × 10^−8^	RNA gene
9q33.1	117,061,772–117,205,684	0.02	6.37	2.96 × 10^−8^	*TRIM32*
10q22.1	69,885,684–69,972,095	0.02	7.52	4.46 × 10^−8^	*COL13A1*
**Locus**	**BP1–BP2 (GRCh 38)**	**F**	**OR**	***p*** **-Value**	**Genes in Sporadic Cecum Cases**
2q12.1	104,421,692–104,699,282	0.01	5.29	1.71 × 10^−8^	9 RNA genes
3q26.33	181,531,075–181,617,660	0.02	4.62	2.63 × 10^−8^	2 RNA genes
4q34.3	179,395,117–179,458,843	0.02	3.99	4.78 × 10^−8^	2 RNA genes
14q21.1	40,125,087–40,268,501	0.14	2.10	3.00 × 10^−9^	4 RNA genes
22q12.1	26,697,195–26,737,148	0.02	3.50	4.80 × 10^−8^	*SEZ6L*
Xq25	127,589,381–128,045,090	0.01	6.69	7.64 × 10^−9^	2 RNA genes
Xp11.22	50,590,556–50,654,091	0.33	1.80	3.42 × 10^−8^	*BMP15*

Abbreviations. Locus, chromosome number with band; BP1 (base pair 1), first position of haplotype with first SNP; BP2 (base pair 2), last position of haplotype with last SNP; F, sample frequency including cases and controls; OR, odds ratio; and Genes, genes spanning the haplotype region.

**Table 2:** Unique statistically significant haplotypes in the analysis of familial and sporadic patients with right-sided colon cancer.

Locus	BP1–BP2 (GRCh 38)	F	OR	*p*-Value	Genes in Familial RCC
1q25.1	175,165,541–175,369,283	0.01	7.12	4.59 × 10^−8^	*KIAA0040*, *TNR*
2q35	214,896,989–215,058,610	0.02	6.72	1.16 × 10^−9^	*ABCA12*
2q13	110,106,331–110,858,564	0.04	4.29	3.65 × 10^−8^	*MALL*, *NPHP1*, *LIMS4*, *RGPD6*, *BUB1*, *ACOXL*
3p24.3	20,080,336–20,158,740	0.02	5.50	3 × 10^−8^	*KAT2B*
3p14.1	68,517,324–68,658,829	0.01	8.68	4.58 × 10^−8^	*TAFA1*
4p14	40,489,769–40,588,891	0.01	7.96	3.75 × 10^−8^	*RBM47*
**Locus**	**BP1–BP2 (GRCh 38)**	**F**	**OR**	***p*** **-Value**	**Genes in Sporadic RCC**
1q24.3	172,445,883–172,711,240	0.02	5.13	1.59 × 10^−8^	*SUCO*, *FASLG*
11q24.3	128,610,263–128,672,508	0.02	4.32	2.86 × 10^−8^	*FLI1*

Abbreviations. Locus, chromosome number with band; BP1 (base pair 1), first position of haplotype with first SNP; BP2 (base pair 2), last position of haplotype with last SNP; F, sample frequency including cases and controls; OR, odds ratio; and Genes, list spanning the haplotype region.

**Table 3:** The clinical features of all the cases used in the study.

Analysis	Gender		Age at Diagnosis		Dukes’ Stage				
	Male	Female	≤50	>50	A	B	C	D	NA
Fam. cecum	41	32	4	69	9	24	23	4	13
Spor. cecum	95	149	10	234	34	100	69	18	23
Fam. right	55	79	6	128	15	55	33	17	14
Spor. right	211	192	17	386	45	156	108	31	63
Fam. left	173	167	23	317	69	89	94	28	60
Spor. left	703	461	69	1095	253	319	314	111	167

Abbreviations. Fam, familial; Spor, sporadic; A, B, C, D, Dukes’ Stages A, B, C and D; NA, data not available.

**Table 4:** (**a**) The haplotypes from Supplementary Table S1 (*p* < 2.5 × 10^−6^). (**b**) The haplotypes from the extended results of Supplementary Table S1.

(**a**)							
**NHAP**	**BP1**	**BP2**	**HAPLOTYPE**	**F**	**OR**	**ST**	***p***
20	216,455,107	216,633,635	GAAGAAAACGAAAGG	0.02	6.48	24	1.11 × 10^−6^
19	216,455,107	216,638,249	GAAGAAAACGAAAGGG	0.01	7.87	24	1.08 × 10^−6^
24	237,175,199	237,257,650	GAGGGGGA**TAGACAG**	0.01	12.1	29	7.88 × 10^−8^
27	237,177,572	237,257,650	AGGGGGA**TAGACAG**	0.01	9.86	28	1.45 × 10^−7^
23	237,177,572	237,266,603	AGGGGGA**TAGACAGA**	0.01	9.44	28	1.39 × 10^−7^
28	237,177,977	237,227,916	GGGGGA**TAGAC**	0.04	4.16	23	2.12 × 10^−6^
27	237,177,977	237,247,063	GGGGGA**TAGACA**	0.02	5.82	24	9.70 × 10^−7^
24	237,177,977	237,257,650	GGGGGA**TAGACAG**	0.01	10.6	31	2.59 × 10^−8^
31	237,194,523	237,257,650	GGGGA**TAGACAG**	0.01	12.3	27	2.32 ×10^−7^
31	237,195,519	237,257,650	GGGA**TAGACAG**	0.01	11.3	26	2.80 × 10^−7^
33	237,197,508	237,257,650	GGA**TAGACAG**	0.01	9.27	25	5.98 × 10^−7^
33	237,199,183	237,257,650	GA**TAGACAG**	0.01	8.64	24	7.83 × 10^−7^
30	237,200,616	237,257,650	A**TAGACAG**	0.01	12.2	28	1.21 × 10^−7^
27	237,200,616	237,266,603	A**TAGACAG**A	0.01	9.55	24	1.12 × 10^−6^
22	237,203,483	237,257,650	**TAGACAG**	0.01	11	32	1.25 × 10^−8^
6	237,212,306	237,227,726	**AGA**	0.08	3.3	23	0.0000013
6	237,212,306	237,227,916	**AGAC**	0.07	3.32	23	1.29 × 10^−6^
18	237,212,306	237,257,650	**AGACAG**	0.02	8.97	29	6.51 × 10^−8^
23	237,212,306	237,266,603	**AGACAG**A	0.01	9.02	25	7.45 × 10^−7^
19	241,326,426	241,344,426	AAGAGCAGA	0.01	11.8	27	2.43 × 10^−7^
19	248,012,296	248,059,856	AAGGGAAGGGAGA	0.01	6.99	26	3.75 × 10^−7^
(**b**)							
**NHAP**	**BP1**	**BP2**	**HAPLOTYPE**	**F**	**OR**	**ST**	***p***
4	237,203,483	237,212,306	TC	0.36	0.58	6	0.00901
4	237,203,483	237,212,306	**TA**	0.51	1.45	4	0.0335
7	237,203,483	237,225,617	**TAG**	0.2	1.89	11	0.00121
7	237,203,483	237,225,617	TCA	0.16	0.47	4	0.0277
9	237,203,483	237,227,726	**TAGA**	0.09	3.21	21	3.85 × 10^−6^
9	237,203,483	237,227,726	TCAA	0.16	0.46	5	0.0255
9	237,203,483	237,227,916	**TAGAC**	0.08	3.25	21	3.65 × 10^−6^
9	237,203,483	237,227,916	TCAAA	0.16	0.48	4	0.032
13	237,203,483	237,247,063	**TAGACA**	0.04	4.18	19	1.38 × 10^−5^
13	237,203,483	237,247,063	**TAGACC**	0.03	2.66	4	0.0268
22	237,203,483	237,257,650	**TAGACAG**	0.02	11	32	1.25 × 10^−8^
22	237,203,483	237,257,650	**TAGAC**CA	0.03	2.6	4	0.0466
22	237,203,483	237,266,603	**TAGAC**CAA	0.01	3.35	4	0.0302
22	237,203,483	237,266,603	TCAAAAGG	0.04	0.08	3	0.0494
26	237,203,483	237,276,068	**TAG**GAAAAGAG	0.01	4.34	4	0.0374
26	237,203,483	237,278,149	**TAG**GAAAAGAGG	0.01	4.32	4	0.0381
2	237,212,306	237,212,306	C	0.41	0.6	7	0.00617
2	237,212,306	237,212,306	**A**	0.59	1.66	7	0.00617
4	237,212,306	237,225,617	**AG**	0.21	1.94	12	0.000531
4	237,212,306	237,225,617	CA	0.21	0.5	5	0.0171
6	237,212,306	237,227,726	**AGA**	0.09	3.3	23	1.30 × 10^−6^
6	237,212,306	237,227,726	CAA	0.21	0.49	5	0.0156
6	237,212,306	237,227,916	**AGAC**	0.08	3.32	23	1.29 × 10^−6^
6	237,212,306	237,227,916	CAAA	0.21	0.51	5	0.0191
11	237,212,306	237,247,063	**AGACA**	0.04	4.24	19	1.22 × 10^−5^
11	237,212,306	237,247,063	**AGAC**C	0.04	3.23	8	0.00437
11	237,212,306	237,247,063	CAAAA	0.18	0.48	5	0.0249
18	237,212,306	237,257,650	**AGACAG**	0.02	8.97	29	6.51 × 10^−8^
18	237,212,306	237,257,650	**AGA**CCG	0.01	4.48	4	0.0354
18	237,212,306	237,257,650	CAAAAG	0.08	0.2	4	0.0399
23	237,212,306	237,266,603	**AGACAG**A	0.02	9.02	25	7.45 × 10^−7^
23	237,212,306	237,266,603	**AGAC**CGG	0.02	3.65	5	0.0241
23	237,212,306	237,266,603	**AGACA**AA	0.01	3.65	4	0.0422
25	237,212,306	237,272,800	**AGACAG**AG	0.01	10.7	20	7.07 × 10^−6^
3	237,225,617	237,227,726	**GA**	0.21	1.67	7	0.00622
3	237,225,617	237,227,916	**GAC**	0.2	1.65	7	0.00786

Abbreviations. NHAP, number of possible haplotypes in this window; BP2, first base pair in haplotype; BP1, last base pair in haplotype; F, frequency; OR, odds ratio; ST, squared T statistics in Wald; and *p*, *p*-value for the test of this in all possible haplotypes in this window.

## Data Availability

The original contributions presented in this study are included in the article/Supplementary Material. Further inquiries can be directed to the corresponding author.

## Supplementary Materials

The following supporting information can be downloaded at https://www.mdpi.com/article/10.3390/ijms27062758/s1.

## Abbreviations

The following abbreviations are used in this manuscript:

CRC	Colorectal cancer
RCC	Right-sided colon cancer
LCC	left-sided colon cancer
GWAS	Genome-wide association study
SNP	Single-nucleotide polymorphism
CIMP	CpG island methylator phenotype
